# The relationship between Tai Chi practice, Psychological Resilience, and depression in older adults: an integrated study of mediation and network analysis

**DOI:** 10.3389/fpubh.2026.1777392

**Published:** 2026-03-04

**Authors:** Faxiang Fan, Hao Gou, Ping Li, Luyao Xiang, Jingtao Du

**Affiliations:** 1 College of Physical Education, Qiannan Normal University for Nationalities, Duyun, China; 2 College of Physical Education, Hunan Normal University, Changsha, China; 3 Zunyi Medical University, Zhuhai, China; 4 College of Sports and Health, Chengdu University of Traditional Chinese Medicine, Chengdu, China

**Keywords:** aging, depression in older adults, mediation effect, psychological network analysis, Psychological Resilience, Tai Chi Exercise

## Abstract

**Background:**

With the increasing global aging population, depression in older adults has become an increasingly prominent issue. As a traditional mind–body exercise, Tai Chi Exercise (TCE) may have the potential to alleviate depression in older adults. However, its underlying mechanisms, particularly the mediating role of Psychological Resilience (PR) and the complex relationships between variables, require systematic exploration.

**Methods:**

This study employed an integrated research design and surveyed 1,143 older adults through questionnaires. Mediation analysis was used to test the mediating effect of PR between TCE and depression (Dep). Additionally, network analysis was used to construct a cross-sectional network model of TCE, PR, and Dep symptoms to reveal the systemic associations between the variables.

**Results:**

Mediation analysis showed that TCE not only negatively predicted Dep directly (*β* = −0.068, *p* < 0.001) but also indirectly alleviated Dep symptoms through improving PR. The indirect effect (−0.192) accounted for 73.88% of the total effect. Network analysis further revealed the complex structure between variables: internal connections within constructs were strong, while direct connections between constructs were weaker. The item “I can achieve my goals” (PR1) was the most influential core node in the network.

**Conclusion:**

This study revealed the dual pathways through which TCE alleviates depression in older adults: the direct effect and the indirect effect through enhancing PR. Network analysis provided systemic-level evidence for this mediating mechanism and identified the key intervention target of “goal achievement.” This suggests that future efforts to promote TCE for enhancing older adults’ mental health should focus on strengthening its role in improving PR, particularly in fostering a sense of personal control and goal achievement, thereby enabling more precise and effective interventions.

## 1 Introduction

With the ongoing acceleration of the global aging process, the proportion of older adults in the social structure is steadily increasing, becoming an undeniable trend in social development (1). This demographic shift poses long-term challenges to the allocation of economic and social resources, and also has profound effects on the health of older adults, especially in terms of the psychological and emotional pressures they face (2). Among various physical and mental health issues in older adults, the widespread presence of depressive moods and their negative impacts are particularly concerning. Depression in older adults not only significantly lowers individual quality of life and social participation willingness, but it may also further exacerbate the decline of physical functions, placing continuous strain on family and social support systems (3, 4). Therefore, within the broader strategy of addressing population aging, focusing on and systematically exploring depression in older adults has become an important research direction for promoting healthy aging and enhancing well-being in later life (5).

### 1.1 The relationship between Tai Chi practice and depression

Tai Chi, rooted in traditional Chinese philosophy and culture, is a form of mind–body exercise that combines slow, fluid movements with breath regulation and focused attention (6). In recent years, Tai Chi has gained widespread popularity and promotion among the older adults due to its gentle, low-energy, adaptable nature, and ease of learning, making it an important choice for daily fitness and recreational activities in this demographic (7, 8). As an integrative mind–body intervention, the benefits of Tai Chi are not only reflected in the physiological aspects, such as improving balance and increasing flexibility, but it is also believed to have a positive regulatory effect on psychological and emotional states (9, 10). The practice of Tai Chi emphasizes the “combination of movement and stillness” and the “nourishment of both body and spirit,” which helps promote relaxation, enhance emotional stability, and may provide inner resources to alleviate negative emotions by enhancing an individual’s sense of self-awareness and control over their environment (11, 12). From a theoretical perspective, the process of practicing Tai Chi itself constitutes a form of mindfulness exercise, which helps practitioners focus their attention on the present moment, reducing rumination and emotional exhaustion, and thereby potentially alleviating symptoms of depression both directly and indirectly (13, 14). In addition, embodied cognition theory posits that bodily states and sensorimotor experiences can shape cognitive–emotional processing; thus, the slow, controlled movements and breath–attention coordination in Tai Chi may foster adaptive emotion regulation and cognitive reappraisal through “embodied” self-regulation, providing a complementary theoretical account for the direct mental-health benefits of TCE (15, 16).” Existing empirical research generally supports this association, with some studies showing that older adults individuals who consistently practice Tai Chi exhibit more positive results in emotional states, life satisfaction, and a reduction in depressive symptoms (15, 16). Although the specific mechanisms of action still require further exploration, both theoretical and empirical evidence suggest that Tai Chi practice may have beneficial effects on depression in the older adults (17). Therefore, this study proposes the following hypothesis: Tai Chi Exercise has a significant impact on depression in the older adults.

### 1.2 The relationship between TCE, PR, and Dep

The potential benefits of TCE on Dep may not follow a single, direct pathway, but rather operate through influencing certain key psychological processes or resources (18). Among these, PR is considered a crucial bridge connecting mind–body practice and emotional health (19). PR typically refers to the psychological traits and dynamic processes that allow individuals to effectively adapt, recover, or even grow when facing stress, adversity, or trauma (20). It is manifested in perseverance when facing difficulties, emotional regulation skills, and a positive interpretation of challenges, all of which play a protective role in maintaining mental health (21). On the one hand, from the general benefits of physical exercise, regular physical activity has been shown to be an effective way to enhance PR (22). The theoretical basis for this is that physical exercise, as a controllable physiological and psychological challenge, can systematically build and strengthen psychological resources by improving an individual’s sense of self-efficacy, promoting positive emotional experiences, and enhancing neuroendocrine regulation (23, 24). Specifically, in the case of TCE, this form of mind–body exercise, which emphasizes the unity of body and mind, gentle and controlled movements, not only provides the aforementioned general benefits but also, due to its unique focus on concentration, coordination, and breathing control, may more directly train individuals’ emotional regulation and stress-coping skills, thus more specifically promoting the development of PR (25–27). Some empirical studies have observed that individuals who engage in mind–body practices like TCE tend to show positive improvements in their level of PR (28). On the other hand, PR itself has a clear protective effect against Dep (29). From a theoretical perspective, individuals with high PR are more likely to cope with life stressors using more adaptive cognitive and behavioral strategies, reducing feelings of helplessness and negative rumination, which in turn lowers the risk of Dep or alleviates its severity (30, 31). A large body of empirical evidence supports that PR is a stable and significant protective factor for Dep, playing a key buffering role between stress and depressive symptoms (32). In conclusion, TCE may not only directly influence Dep but may also, through cultivating and enhancing PR as an internal psychological resource, indirectly provide a more profound and lasting relief for Dep (33, 34). Therefore, this study further hypothesizes: PR mediates the relationship between TCE and Dep in older adults.

**Figure 1:**
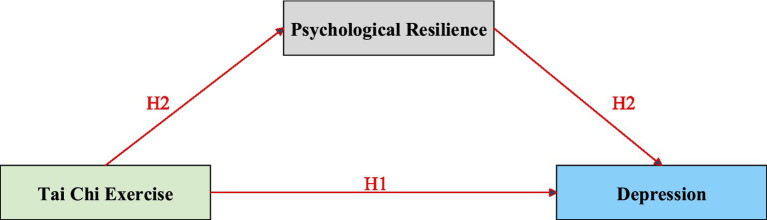
Hypothesized research model illustrating the direct (TCE → Dep) and indirect (TCE → PR → Dep) relationships among TCE, PR, and Dep.

Taken together, mindfulness and embodied cognition provide a coherent mind–body account of why TCE may influence Dep both directly and indirectly. As an “embodied mindfulness” practice, Tai Chi repeatedly trains present-moment attention and breath–movement synchronization, which can reduce maladaptive rumination and improve emotion regulation; simultaneously, embodied cognition emphasizes that these sensorimotor experiences can reshape affective-cognitive patterns and strengthen perceived control. These self-regulatory gains may translate into broader psychological resources—namely PR—because repeated mastery experiences and enhanced self-regulation support adaptive coping when facing stressors. In turn, PR functions as a protective factor that buffers stress-related negative affect and cognitive vulnerability, thereby lowering the risk or severity of Dep. Based on this integrated rationale, we propose that TCE negatively predicts Dep (H1) and that PR partially mediates the association between TCE and Dep (H2), as illustrated in Figure 1.

### 1.3 Deepening the exploration of variable relationships using network analysis

To further understand the complex interactive relationships between TCE, PR, and Dep in older adults, this study introduces the emerging method of psychological network analysis to provide an integrated exploration, building on traditional variable relationship analysis (35, 36). Network analysis is a modeling approach that views psychological constructs as interconnected nodes and estimates the unique connections between these nodes to reveal the overall psychological structure (37). Its core advantage lies in the fact that it does not rely on latent common factor assumptions, but directly models psychological phenomena as dynamic, observable systems of interaction between variables, thereby allowing for the direct visualization of associations, indirect paths, and the most influential core nodes within the network (38, 39). Compared to traditional methods that focus solely on means and causal paths, network analysis can more finely depict the parallel and potentially conditional relationships between variables, helping to identify the key leverage points and potential maintenance mechanisms that influence the entire psychological state (40, 41). Specifically, in this study, placing TCE, PR, and the various dimensions of Dep symptoms into a single network model for analysis will not only validate the existence of mediation pathways but also further reveal: which specific Dep symptoms are most closely related to TCE or PR; which aspects of PR may occupy central positions within the network; and whether there are key bridges within the system that directly link mind–body exercise with emotional improvement (42–45). This analytical strategy is expected to go beyond a general understanding of the overall relationships between variables and, from a systemic perspective (46), identify the most efficient and targeted entry points for interventions on Dep in older adults, providing more refined theoretical guidance for the design of precise and effective TCE interventions (47, 48).

To further clarify the purpose of the present study, we formulate the following specific research objectives: (1) to examine whether Tai Chi Exercise (TCE) is associated with lower depression (Dep) among older adults (corresponding to H1); (2) to test whether Psychological Resilience (PR) explains this association as a mediating mechanism (corresponding to H2); and (3) to apply psychological network analysis to depict item-/symptom-level connections among TCE, PR, and Dep, thereby identifying influential or potentially bridging nodes that may inform more precise intervention targets in future Tai Chi–based mental health programs.

## 2 Methods

### 2.1 Participants and procedures

From January to June 2025, a random sampling method was used to select 1,300 participants from Tai Chi associations and senior universities in Guiyang (Guizhou Province), Liuzhou and Nanning (Guangxi Zhuang Autonomous Region) as survey subjects. These three cities were selected because they are typical urban areas in Southwest China with well-established Tai Chi associations and senior universities, which provided a stable sampling frame for recruitment; moreover, including one provincial capital and two major prefecture-level cities helps increase geographic and socio-economic diversity, thereby strengthening the representativeness of the research design. A total of 1,229 paper questionnaires were distributed, and after excluding 86 invalid questionnaires, 1,143 valid questionnaires were returned, with a valid return rate of 93%. The criteria for invalid questionnaires included excessive missing answers, incomplete responses, and patterned answers (e.g., selecting the same option continuously). After excluding invalid questionnaires due to patterned responses and failure to pass lie detection questions, missing values in the remaining data were handled using the Multiple Imputation by Chained Equations (MICE) method. The imputation operation was carried out using the mice package in the R programming environment (49).

**Figure 2:**
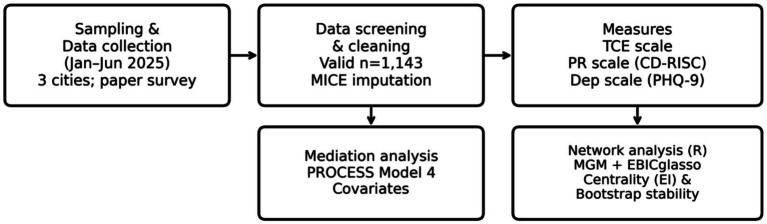
Research design and analysis workflow of the study.

The overall research design and analysis workflow are summarized in Figure 2.

A total of 1,143 older adults individuals were included in the study, consisting of 466 males (40.80%) and 677 females (59.20%). The education levels were as follows: illiterate or semi-illiterate 467 people (40.90%), primary school 322 people (28.20%), junior high school 227 people (19.90%), and high school or above 127 people (11.10%). The age distribution was: 637 people (55.70%) aged 60–70, 332 people (29.00%) aged 71–80, and 174 people (15.20%) aged 80 and above. Regarding marital status, 760 people (66.50%) had a spouse, while 383 people (33.50%) were without a spouse. The detailed distribution is shown in Table 1.

**Table 1:** Demographic characteristics of the participants in the survey (*n* = 1,143).

Variable	Sort	Frequency	Scale
Sex	Male	466	40.80%
	Female	677	59.20%
Education level	Illiterate or semi-illiterate	467	40.90%
	Primary school	322	28.20%
	Middle school	227	19.90%
	High school or above	127	11.10%
Age	60–70 years	637	55.70%
	71–80 years	332	29.00%
	Over 80 years	174	15.20%
Marital status	With a spouse	760	66.50%
	Without a spouse	383	33.50%

### 2.2 Tai Chi Exercise Scale

The Tai Chi Exercise Intensity Scale is adapted from Liang’s (50) “Physical Activity Intensity Scale” and designed in combination with the characteristics of fitness Qigong. The scale includes questions such as: “How long is your Tai Chi practice session each time?” and “How often do you practice Tai Chi?” The aim is to accurately measure the exercise intensity of the participants’ Tai Chi practice. The scale evaluates three aspects: exercise intensity, duration, and frequency, with the total exercise volume calculated as the product of these three factors. The scoring criteria are as follows: ≤19 points indicate low exercise intensity, 20–42 points indicate moderate exercise intensity, and ≥43 points indicate high exercise intensity. In this study, the Cronbach’s alpha coefficient of the scale was 0.803.

### 2.3 Psychological Resilience Scale

The Psychological Resilience Scale, developed by Connor et al. and revised into a Chinese version by Yu and Zhang, contains 25 items (51). Each item is rated on a Likert 5-point scale, ranging from 1 (“Not at all like me”) to 5 (“Almost always like me”). A higher score indicates stronger Psychological Resilience in the individual. In this study, the Cronbach’s alpha coefficient for this scale was 0.910, with Cronbach’s alpha values ranging from 0.814 to 0.930 for its dimensions. Confirmatory factor analysis showed that the model fit indices were within an acceptable range (*χ*^2^/df = 1.601, RMSEA = 0.023, SRMR = 0.018, CFI = 0.988, TLI = 0.986).

### 2.4 Depression Scale

The PHQ-9 scale is used to assess depressive symptoms (52). It contains 9 items, rated on a 4-point Likert scale, with a total score range of 0 to 27. A higher score indicates more severe depressive symptoms. In this study, the Cronbach’s alpha coefficient for this scale was 0.877. Confirmatory factor analysis showed that the model fit indices were within an acceptable range (*χ*^2^/df = 1.543, RMSEA = 0.025, SRMR = 0.016, CFI = 0.995, TLI = 0.993).

### 2.5 Data analysis

Mediation analysis was conducted using SPSS 27.0 and PROCESS macro (Model 4) on 1,143 valid questionnaires, with gender, age, marital status, and education level (53). Network analysis was performed in R 4.3.2, using mixed graphical models for estimation and the EBICglasso algorithm for regularization to simplify the network structure (54). The hyperparameter *γ* of the Extended Bayesian Information Criterion (EBIC) was set to 0.5. For assessing node centrality, expected influence was chosen as the measure of node impact, which has demonstrated good stability when dealing with mixed polarity networks. Finally, 1,000 iterations of bootstrapping were performed to calculate the 95% confidence intervals for edge weights, assess the accuracy of estimates, and test the stability of centrality indicators. An acceptable stability coefficient (CS) was set at CS > 0.25, with CS > 0.5 indicating good stability (55).

## 3 Results

### 3.1 Common method bias test

Harman’s single-factor test was used to check for common method bias in the data. The results showed that the cumulative variance contribution of the first factor was 29.091%, which is below the recommended threshold of 40%, indicating no significant common method bias in the data.

### 3.2 Descriptive statistics and correlation analysis

The results showed a significant positive correlation between TCE and PR (*p* < 0.001), and significant negative correlations between TCE, PR, and Dep (*p* < 0.001). See Table 2.

**Table 2:** Means, standard deviations, and correlation coefficients of all variables.

Variables	M ± SD	Tai Chi Exercise	Psychological Resilience	Depression
Tai Chi Exercise	22.54 ± 28.18	1		
Psychological Resilience	2.93 ± 0.78	0.370**	1	
Depression	1.59 ± 0.79	−0.259**	−0.538**	1

*p < 0.05, **p < 0.01, ***p < 0.001.

### 3.3 Mediation analysis

The study used TCE as the *X* variable, PR as the *M* variable, and Dep as the *Y* variable, and employed the PROCESS V4.1 mediation model plugin in SPSS 27.0 to test the mediating effect of PR. The results are shown in Table 3. Gender, age, marital status, and education level were included in the model as covariates. TCE significantly negatively predicted Dep (*β* = −0.068, *p* < 0.001); TCE significantly positively predicted PR (*β* = 0.371, *p* < 0.001); and PR significantly negatively predicted Dep (*β* = −0.517, *p* < 0.001). Further analysis showed that after adding PR as a mediator, TCE still significantly negatively predicted Dep (*β* = −0.260, *p* < 0.001). Bootstrap testing revealed that the indirect effect of PR was −0.192, with a 95% confidence interval that did not include zero, confirming the statistical significance of the mediating effect, as shown in Table 4. This effect explained 73.88% of the total effect.

**Table 3:** Tai Chi Effects on resilience and depression.

Dependent variable	Independent variable	*R*	*R^2^*	*F*(df)	*β*	*t*
Psychological Resilience	Tai Chi Exercise	0.394	0.155	41.660	0.371	13.607
	Sex				−0.107	−1.895
	Age				0.087	1.265
	Education level				0.054	1.940
	Marital status				0.110	1.839
Depression	Tai Chi Exercise	0.543	0.295	79.277	−0.068	−2.524
	Psychological Resilience				−0.517	−19.068
	Sex				0.012	0.229
	Age				0.034	0.957
	Education level				−0.004	−0.174
	Marital status				0.065	1.186

**Table 4:** Total, direct, and indirect effects.

Effect	Effect size	Bootstrap standard error	95% lower	95% upper	Effect size (%)
Total	−0.260	0.029	−0.316	−0.204	
Direct	−0.068	0.027	−0.121	−0.015	26.12%
Indirect	−0.192	0.016	−0.224	−0.161	73.88%

### 3.4 Network analysis

**Figure 3:**
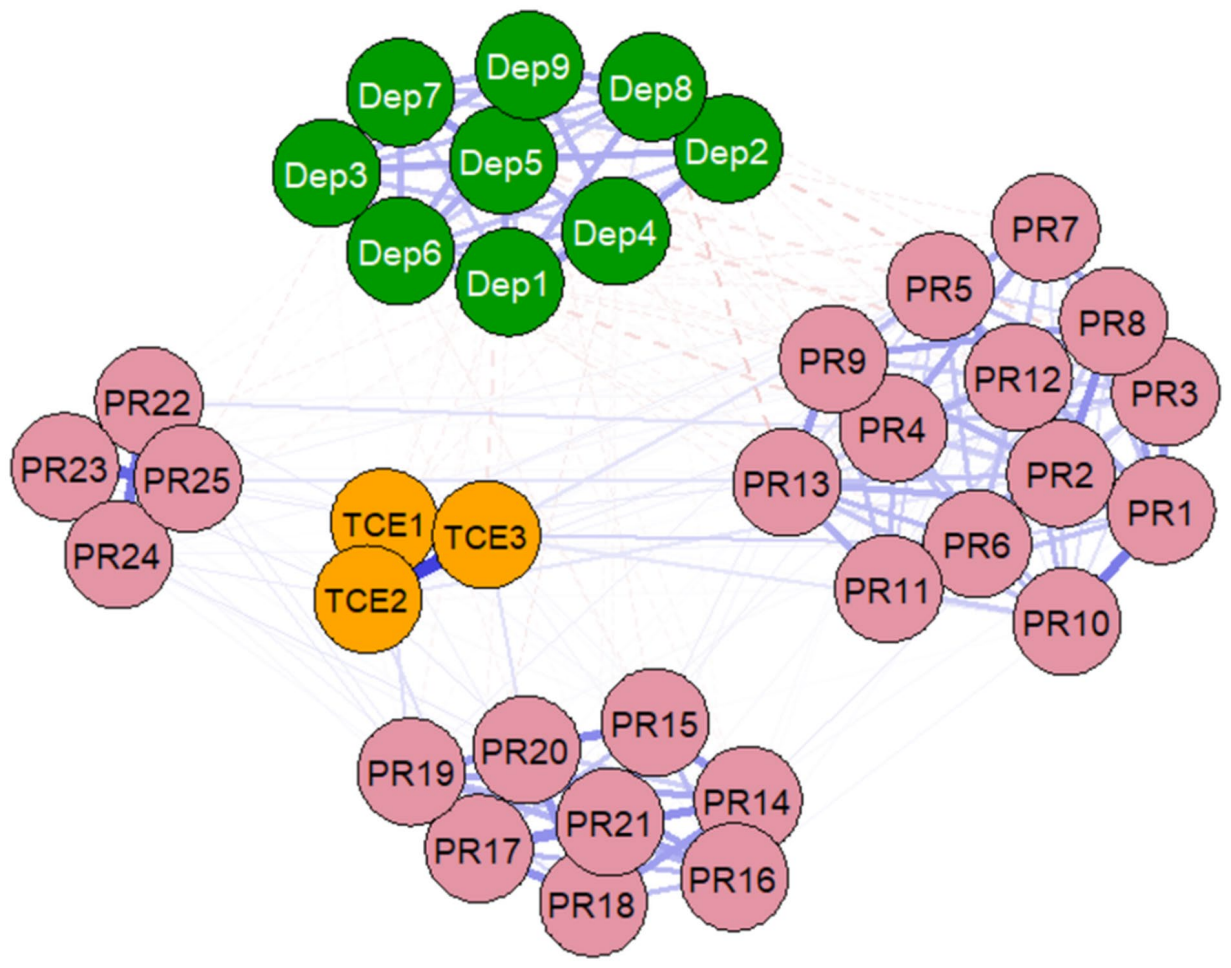
Cross-sectional network structure of Tai Chi Exercise (TCE), Psychological Resilience (PR), and Depression Symptoms (Dep).

According to the results of the cross-sectional network analysis (see Figure 3, Supplementary Table S-2), the overall network composed of all items from TCE, PR, and Dep exhibited a moderately dense relational structure, with a network density of 0.4505 and 300 non-zero edges (45.05% of all possible connections). Among all the associations, the three most prominent connections displayed clear patterns: first, the strongest connection occurred within the same construct, where the positive correlation between the duration of a single Tai Chi session (TCE1) and exercise frequency (TCE2) was the most significant (edge weight = 0.343), reflecting a high degree of internal consistency within the scale. Second, the most representative cross-network connection, i.e., the direct association between items from different core constructs, was significantly weaker than the internal associations within the scale. Among these, a notable negative connection was found between the PR item “I enjoy challenges” (PR13) and the Dep item “Others notice that I move or speak slowly, or on the contrary, I am more active than usual” (Dep8) (weight = −0.064). Third, within the PR network, there were several moderate-strength connections, such as the strong positive correlation between the items “I can adapt to changes” (PR14) and “Dealing with stress makes me feel powerful” (PR16) (weight = 0.220). Overall, the edge weights of the direct cross-network connections were generally small, suggesting that the direct pairwise associations between TCE, PR, and Dep symptoms are relatively weak. The intrinsic relationships among these variables may rely more on potential indirect paths or overall system interactions, which provides preliminary network-level evidence for subsequent testing of the mediating role of PR.

**Figure 4:**
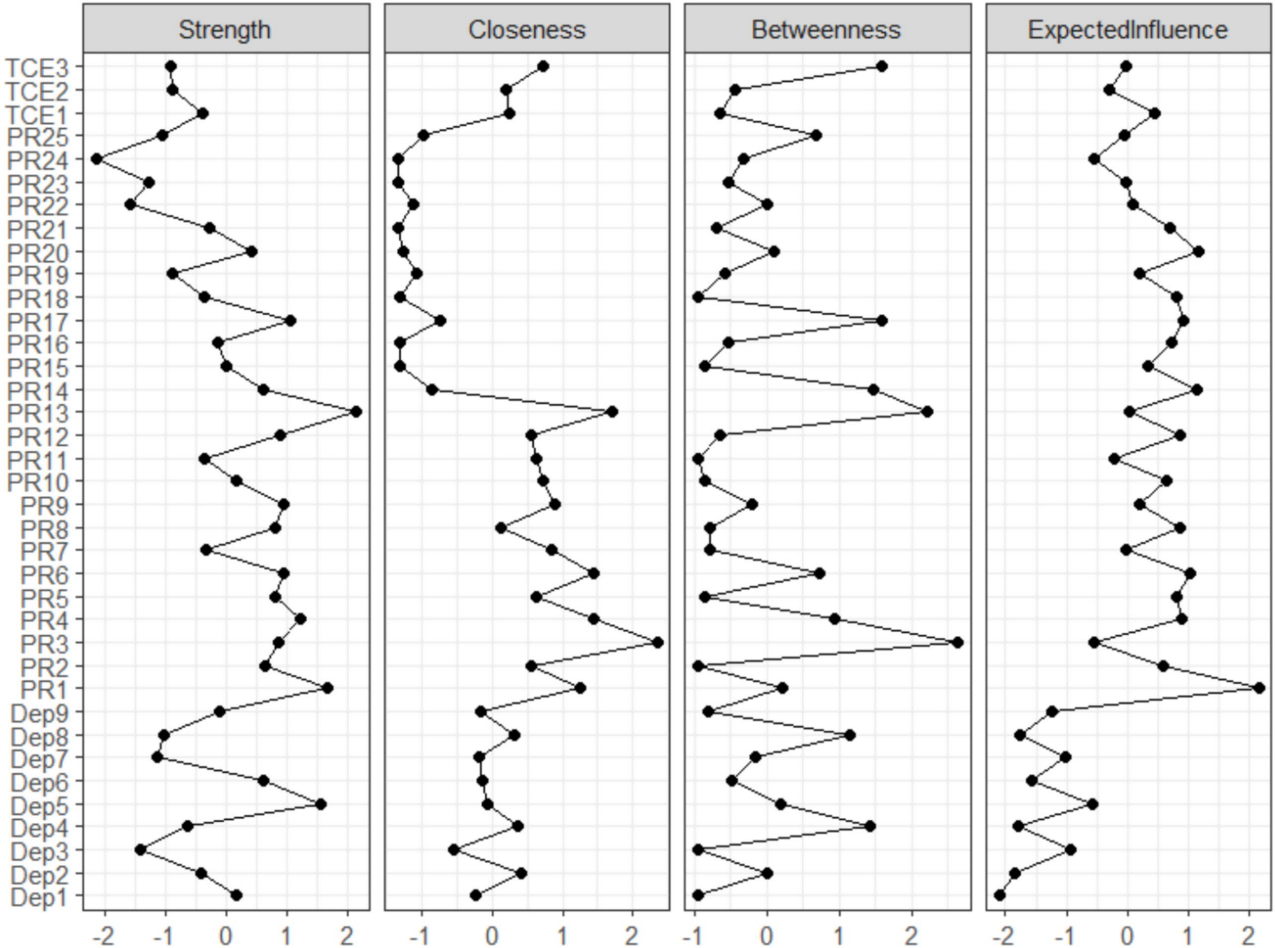
Distribution of expected influence centrality values across network nodes.

To identify key influential core nodes in the network, this study used expected influence centrality as the analysis metric. Expected influence centrality not only calculates the sum of the absolute values of all node connections but also retains the positive and negative signs of these connections. This allows for a more accurate reflection of how a node’s state change may impact the rest of the network, especially in a psychological network with mixed positive and negative associations. Compared to traditional metrics like degree centrality, expected influence centrality typically shows better stability when there are fluctuations in network estimates. Based on the stability test results from Figure 4 and the specific expected influence values (see Supplementary Table S-3), the analysis revealed significant differences in centrality among the nodes. Among them, the PR item “I can achieve my goals” (PR1) had the highest expected influence value (EI = 2.149), significantly higher than other nodes. This indicates that PR1 occupies the most central position in the overall network constructed in this study, and its positive or negative changes are expected to have the most significant radiating effects on other variables in the network, including other PR items, depressive symptoms, and even exercise behavior.

To evaluate the reliability of the constructed network, this study conducted a stability test based on bootstrapping. The results indicated that the 95% bootstrap confidence intervals for the edge weights in the network were overall quite narrow (see Supplementary Figure S-2), suggesting that the network structure has acceptable stability. Specifically, the related stability coefficient for the expected influence centrality was 0.75, reaching the highest level of the test, indicating that this centrality metric is stable and reliable within the network. The stability of other metrics, such as distance and edge weights, also showed good performance.

## 4 Discussion

Building on our research questions and the conceptual model (Figure 1), this study examined whether Tai Chi Exercise (TCE) is associated with depression (Dep) in older adults (H1) and whether Psychological Resilience (PR) serves as an explanatory mechanism (H2). Overall, our findings support both hypothesized pathways: TCE is negatively associated with Dep, and PR partially mediates the relationship between TCE and Dep. Interpreting these results through the integrated framework presented in the Introduction, the direct pathway can be understood in terms of mindfulness- and embodiment-based self-regulation, whereas the indirect pathway reflects the role of PR as a key psychological resource that buffers depressive vulnerability.

### 4.1 The impact of Tai Chi Exercise (TCE) on depression in older adults

The results of this study confirm that TCE has a significant negative predictive effect on depressive symptoms in older adults, supporting the first core hypothesis of the study (56). This finding aligns with the conclusions of several existing studies, which suggest that regular participation in Tai Chi can effectively improve the emotional state of older adults and reduce depression levels (57, 58). From a theoretical perspective, this association is strongly supported by embodied cognition theory (59). This theory emphasizes that the body’s movement state, perceptual experiences, and cognitive-emotional processes are not separate, but are deeply integrated and mutually shaping (60). Tai Chi, as a typical “embodied” practice, involves slow, fluid movements that require focused attention (61). It is not only a physical activity but also constitutes a unique emotional regulation and cognitive restructuring process (62). Through the coordination of “adjusting the body,” “adjusting the breath,” and “adjusting the mind,” practitioners may directly break the vicious cycle of bodily sluggishness, negative rumination, and emotional distress commonly associated with depression, promoting positive changes in both physical and mental states (63–65). When considered in the context of Chinese culture, Tai Chi is deeply rooted in traditional culture, with high popularity and acceptance, offering older adults a non-pharmacological intervention option with low psychological barriers and high cultural identity (66–68). The group practice format of Tai Chi often naturally incorporates elements of social interaction and collective support, which may further enhance its positive effect in alleviating feelings of loneliness (69), improving life meaning, and resisting depression (70, 71). Therefore, this study empirically validates that Tai Chi, as an activity that aligns with the physical and mental characteristics and cultural background of older adults, has clear value in promoting their psychological well-being (72, 73).

### 4.2 The mediating role of Psychological Resilience (PR)

The results of the mediation analysis in this study show that PR plays a significant partial mediating role between TCE and depression in older adults, thus validating the research hypothesis (19). This finding reveals the internal psychological mechanism through which Tai Chi alleviates depression, namely that its benefits are partly achieved by cultivating and enhancing the core psychological resource of PR (74, 75). On one hand, the promoting effect of TCE on PR can be explained from the perspective of self-efficacy theory (76). This theory suggests that an individual’s belief in their ability to successfully perform a behavior and achieve a specific outcome is a key driver of psychological motivation (77). The practice of Tai Chi requires practitioners to continually learn and master a series of complex mind–body coordination movements (78). This progressive experience of success significantly enhances the individual’s sense of self-efficacy in areas such as body control and stress coping (79). At the same time, as a practice that emphasizes inner awareness and balance, Tai Chi helps practitioners develop better acceptance and regulation of their emotional and cognitive states (80). These improvements in control, sense of achievement, and emotional regulation, directly brought about by the exercise, provide a solid foundation for the development of PR, thereby enhancing individuals’ adaptability and recovery ability when facing adversity (81, 82). On the other hand, PR itself has a clear protective effect against depression (83). According to the protective factor model of PR, individuals with high PR tend to have a more positive cognitive style, stronger problem-solving abilities, and richer social support resources (84, 85). These protective factors effectively buffer the negative emotional impacts of stress events, reducing susceptibility to depression (86). A large body of empirical research consistently shows that PR is a stable negative predictor of depressive symptoms and plays a key regulatory role between stress and mental health (87). In conclusion, this study shows that TCE not only directly benefits the emotional health of older adults, but more importantly, it indirectly alleviates depression through the systematic enhancement of PR—boosting individuals’ internal strength and resilience in facing challenges (88). This suggests that future Tai Chi intervention programs aimed at promoting the psychological health of older adults should consciously incorporate the cultivation of PR elements to maximize intervention benefits (89, 90).

### 4.3 Complex association patterns revealed by network analysis

The results of the network analysis provide a unique systems perspective for understanding the complex relationships between TCE, PR, and Dep (28). First, the strongest connections in the network occur within the individual scales, such as the strong correlations between TCE items. From a psychometric perspective, this confirms that the research tools used have good internal consistency, reflecting the high degree of correlation among different observational indicators within the same psychological construct, thus ensuring the reliability of subsequent analyses of the relationships between variables (91). More importantly, the direct connections across the network (e.g., between TCE and Dep, PR and Dep) are generally weak, and this finding has significant theoretical and empirical implications (92). According to network psychology theory, dimensions of mental health are typically organized in a modular manner, and modules with weaker direct associations tend to influence each other through “bridge” nodes or indirect path s (93). The findings of this study support this structure, indicating that the relationship between TCE and depressive symptoms is not simply a direct antagonistic one (94). Its positive effects are more likely to be realized indirectly by cultivating a more adaptive psychological system (such as the PR network) (95). For example, the strong connection between “adapting to changes” and “drawing strength from stress” within the PR network (PR14-PR16) reveals a core psychological mechanism for positive coping, which may be a key pathway through which Tai Chi practitioners convert the sense of control from physical exercise into general psychological resources (11, 96). Additionally, the negative connection between “enjoying challenges” and “mental/physical agitation/sluggishness” (PR13-Dep8) specifically demonstrates the potential mitigating effect of positive psychological traits on certain behavioral symptoms (11, 97). Therefore, the network analysis not only structurally validates the reasonableness of the mediation analysis but also more precisely depicts the specific pathways of action within and between the variables, emphasizing the importance of future integrative intervention strategies targeting key symptoms or traits, with a core focus on promoting PR (98, 99).

### 4.4 Identification of core network nodes and implications

The core node revealed by the network analysis—PR1 (“I can achieve my goals”)—has the highest expected influence. This finding can be deeply interpreted from both theoretical and practical perspectives (100, 101). From a theoretical standpoint, it strongly resonates with the core concepts of goal-setting theory and self-regulation theory (102). These theories argue that clear and committed goals are key internal drivers of individual behavior, maintaining cognitive engagement and emotional stability (77). For older adults, whether it is sticking with a long-term exercise like Tai Chi or dealing with the physiological and social challenges of daily life, the belief in “I can achieve my goals” serves as the fundamental source of agency (19). It is not an isolated self-feeling, but a metacognitive hub that initiates and sustains the positive adaptation process: the enhancement of this belief may positively stimulate the individual’s willingness and persistence in exercise participation (linked to the TCE node), while systematically organizing other Psychological Resilience resources (such as persistence PR2 and focus PR4), and effectively inhibiting core depressive symptoms such as helplessness and disappointment (e.g., Dep6) (103–105). In the context of positive aging in China, the belief in one’s self-efficacy to achieve goals is particularly significant in combating the sense of “worthlessness” that may arise from changes in social roles and physical decline (106, 107). Therefore, the central position of PR1 in the network not only identifies a high-leverage intervention target methodologically but also suggests theoretically that when promoting the mental health of older adults through mind–body practices such as Tai Chi, psychological components such as goal management and the accumulation of small successes should be deliberately integrated to strengthen their core belief in “goal achievement.” This could potentially leverage the entire psychological adaptation system to evolve toward a more positive and resilient direction (9, 78, 108, 109).

### 4.5 Limitations and future directions

This study has several limitations, which future research can address. First, although the cross-sectional design revealed associations and mediation mechanisms between variables, it cannot strictly infer causal relationships (110, 111). Future studies could use longitudinal tracking or randomized controlled trial designs to more clearly elucidate the temporal and causal links between Tai Chi Exercise, the enhancement of Psychological Resilience, and the alleviation of depressive symptoms (112). Second, the sample primarily came from community Tai Chi associations and senior universities, which may have overestimated the engagement of exercisers and their psychological resources (113). Future research should expand the sampling scope to include a broader and more representative population of older adults from various communities, including individuals who participate less in organized activities, to improve the external validity of the research findings (114). Furthermore, the study relied mainly on self-report questionnaires, which may be subject to common method bias and social desirability effects (115). Future studies could combine objective physiological indicators (e.g., heart rate variability, cortisol levels), behavioral records, and informant reports for multimodal assessments, thus enhancing the ecological validity and accuracy of the measurements (116, 117). Finally, while network analysis provides a detailed depiction of the complex interactive structure between variables, it is inherently based on correlations. Future research could further explore network-oriented interventions by experimentally manipulating the core nodes in the network (e.g., specifically enhancing the “goal achievement” belief) to empirically test whether these connections have causal effects (117–119). This could provide stronger evidence for developing precision psychological intervention programs based on network theory (120).

## 5 Conclusion

This study systematically explored the relationship between Tai Chi Exercise (TCE), Psychological Resilience (PR), and depression in older adults by integrating mediation analysis and network analysis methods. The results indicate that TCE not only directly alleviates depressive symptoms in older adults but also produces a deeper indirect protective effect by effectively enhancing their core psychological resource, PR. Network analysis further reveals, from a systems perspective, that PR—particularly the belief in “goal achievement”—serves as a key hub connecting mind–body exercise and emotional health. The direct associations between variables are relatively weak, providing structural support for the existence of a mediation pathway. These findings not only empirically validate the dual pathways through which TCE promotes mental health but also deepen our understanding of the psychological adaptation mechanisms in older adults. Practically, this study suggests that future health promotion programs for older adults, when promoting Tai Chi as a traditional mind–body exercise, should focus on integrating elements that cultivate Psychological Resilience, particularly enhancing their sense of goal achievement and control. This approach will more precisely and effectively maintain and improve the psychological health of the older population.

## Data Availability

The original contributions presented in the study are included in the article/Supplementary material, further inquiries can be directed to the corresponding authors.
