# Supplementary material for: The relationship between Tai Chi practice, Psychological Resilience, and depression in older adults: an integrated study of mediation and network analysis
Source: Front Public Health. 2026 Mar 4;14:1777392. doi: 10.3389/fpubh.2026.1777392 (PMC12995767; doi:10.3389/fpubh.2026.1777392)
Supplement: Supplementary file 1 [file Table_1.docx]

Supplementary Material

# Supplementary Figures and Tables

## Supplementary Tables

**Table S-1** Descriptive Statistics for Tai Chi Exercise, Psychological Resilience, and Depression Items

| Variable | Item | Label | M | SD |
| --- | --- | --- | --- | --- |
| Tai Chi Exercise | How long do you practice Tai Chi each time? | TCE1 | 2.730 | 1.423 |
|  | How often do you practice Tai Chi? | TCE2 | 2.841 | 1.448 |
|  | What is the intensity of your Tai Chi practice? | TCE3 | 2.752 | 1.462 |
| Psychological Resilience | I am able to achieve my goals. | PR1 | 2.837 | 1.412 |
|  | When things seem hopeless, I do not give up easily. | PR2 | 2.905 | 1.388 |
|  | I know where to seek help. | PR3 | 2.890 | 1.380 |
|  | I can concentrate and think clearly under pressure. | PR4 | 2.927 | 1.371 |
|  | I enjoy taking the lead in solving problems. | PR5 | 2.899 | 1.374 |
|  | I do not get discouraged by failure. | PR6 | 2.906 | 1.383 |
|  | I think of myself as a strong person. | PR7 | 2.871 | 1.369 |
|  | I can make unconventional or difficult decisions. | PR8 | 2.915 | 1.391 |
|  | I can handle unpleasant emotions. | PR9 | 2.916 | 1.376 |
|  | I have to act on my intuition sometimes. | PR10 | 2.906 | 1.359 |
|  | I have a strong sense of purpose. | PR11 | 2.864 | 1.381 |
|  | I feel in control of my life. | PR12 | 2.946 | 1.381 |
|  | I enjoy challenges. | PR13 | 2.935 | 1.391 |
|  | I can adapt to change. | PR14 | 2.886 | 1.442 |
|  | Past successes give me confidence to face challenges. | PR15 | 2.953 | 1.374 |
|  | Dealing with stress makes me feel powerful. | PR16 | 2.926 | 1.381 |
|  | After going through difficulties or illness, I tend to recover quickly. | PR17 | 2.946 | 1.372 |
|  | Things happen for a reason. | PR18 | 2.982 | 1.375 |
|  | No matter the outcome, I always do my best. | PR19 | 2.959 | 1.374 |
|  | I work hard to achieve my goals. | PR20 | 2.967 | 1.371 |
|  | I am proud of my achievements. | PR21 | 2.979 | 1.374 |
|  | I have close, secure relationships. | PR22 | 2.952 | 1.433 |
|  | Sometimes, fate or God can help. | PR23 | 2.990 | 1.423 |
|  | No matter what happens, I can cope. | PR24 | 2.983 | 1.461 |
|  | I can see the humorous side of things. | PR25 | 3.022 | 1.419 |
| Depression | I feel bored doing anything, or I don't want to do anything at all. | Dep1 | 1.640 | 1.108 |
|  | I feel depressed, hopeless, or despairing. | Dep2 | 1.564 | 1.112 |
|  | I have difficulty falling asleep; wake up in the middle of the night, or on the contrary, sleep too much. | Dep3 | 1.605 | 1.099 |
|  | I feel tired or lack energy. | Dep4 | 1.585 | 1.122 |
|  | I have a poor appetite or overeat. | Dep5 | 1.543 | 1.119 |
|  | I don't like myself—I feel I am not doing well, disappointed in myself, or have negative family expectations. | Dep6 | 1.597 | 1.083 |
|  | I find it difficult to concentrate, such as reading the newspaper or watching TV. | Dep7 | 1.592 | 1.100 |
|  | Others notice I am slow to act or speak; or conversely, I am more active than usual—restless, unable to sit still. | Dep8 | 1.634 | 1.092 |
|  | I think it would be better if I were dead or think about self-harm. | Dep9 | 1.591 | 1.122 |

**Table S-2** Cross-Sectional Network Edge Weights (Top 30)

| Rank | Node |  | Node | Correlation Coefficient |
| --- | --- | --- | --- | --- |
| 1 | TCE1 | - | TCE2 | 0.343 |
| 2 | TCE1 | - | TCE3 | 0.336 |
| 3 | PR22 | - | PR25 | 0.272 |
| 4 | PR22 | - | PR23 | 0.267 |
| 5 | TCE2 | - | TCE3 | 0.255 |
| 6 | PR24 | - | PR25 | 0.248 |
| 7 | PR23 | - | PR24 | 0.246 |
| 8 | PR14 | - | PR16 | 0.220 |
| 9 | PR23 | - | PR25 | 0.204 |
| 10 | PR22 | - | PR24 | 0.179 |
| 11 | PR1 | - | PR10 | 0.177 |
| 12 | Dep2 | - | Dep8 | 0.160 |
| 13 | PR15 | - | PR20 | 0.157 |
| 14 | PR20 | - | PR21 | 0.153 |
| 15 | PR14 | - | PR17 | 0.148 |
| 16 | PR2 | - | PR8 | 0.147 |
| 17 | PR17 | - | PR18 | 0.146 |
| 18 | PR17 | - | PR21 | 0.146 |
| 19 | PR17 | - | PR19 | 0.144 |
| 20 | PR14 | - | PR15 | 0.142 |
| 21 | PR18 | - | PR20 | 0.140 |
| 22 | PR15 | - | PR18 | 0.138 |
| 23 | PR5 | - | PR12 | 0.137 |
| 24 | Dep3 | - | Dep6 | 0.135 |
| 25 | Dep5 | - | Dep9 | 0.134 |
| 26 | Dep1 | - | Dep4 | 0.134 |
| 27 | Dep5 | - | Dep7 | 0.133 |
| 28 | PR19 | - | PR20 | 0.132 |
| 29 | PR9 | - | PR13 | 0.132 |
| 30 | PR1 | - | PR3 | 0.132 |

**Table S-3** Expected Influence Scores for Each Node in the Network

| Rank | Node | Expected Influence |
| --- | --- | --- |
| 1 | TCE1 | 0.452 |
| 2 | TCE2 | -0.287 |
| 3 | TCE3 | -0.035 |
| 4 | PR1 | 2.149 |
| 5 | PR2 | 0.596 |
| 6 | PR3 | -0.553 |
| 7 | PR4 | 0.878 |
| 8 | PR5 | 0.806 |
| 9 | PR6 | 1.014 |
| 10 | PR7 | -0.012 |
| 11 | PR8 | 0.864 |
| 12 | PR9 | 0.190 |
| 13 | PR10 | 0.646 |
| 14 | PR11 | -0.211 |
| 15 | PR12 | 0.848 |
| 16 | PR13 | 0.035 |
| 17 | PR14 | 1.140 |
| 18 | PR15 | 0.348 |
| 19 | PR16 | 0.716 |
| 20 | PR17 | 0.925 |
| 21 | PR18 | 0.806 |
| 22 | PR19 | 0.196 |
| 23 | PR20 | 1.154 |
| 24 | PR21 | 0.691 |
| 25 | PR22 | 0.097 |
| 26 | PR23 | -0.017 |
| 27 | PR24 | -0.550 |
| 28 | PR25 | -0.043 |
| 29 | Dep1 | -2.104 |
| 30 | Dep2 | -1.854 |
| 31 | Dep3 | -0.942 |
| 32 | Dep4 | -1.793 |
| 33 | Dep5 | -0.565 |
| 34 | Dep6 | -1.576 |
| 35 | Dep7 | -1.022 |
| 36 | Dep8 | -1.760 |
| 37 | Dep9 | -1.228 |

## Supplementary Figures


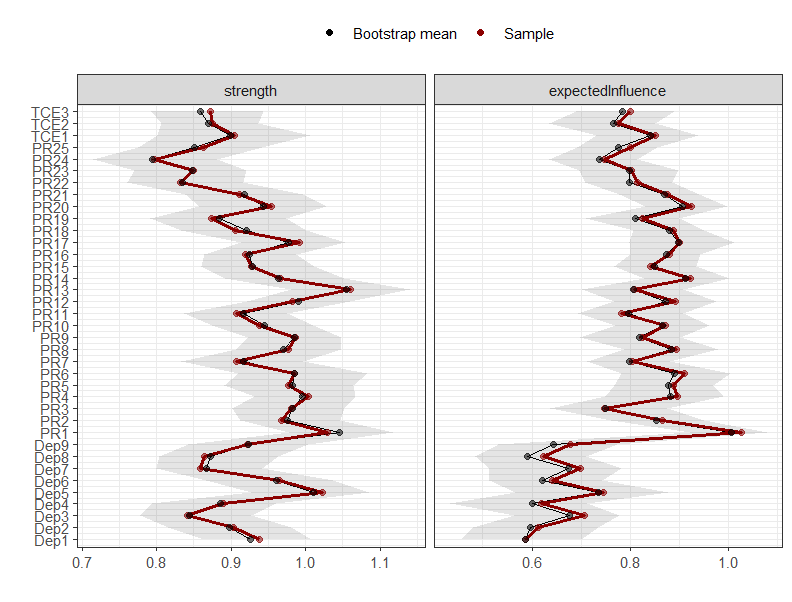


**Figure S-1** Stability Analysis Results (Part 1)

**
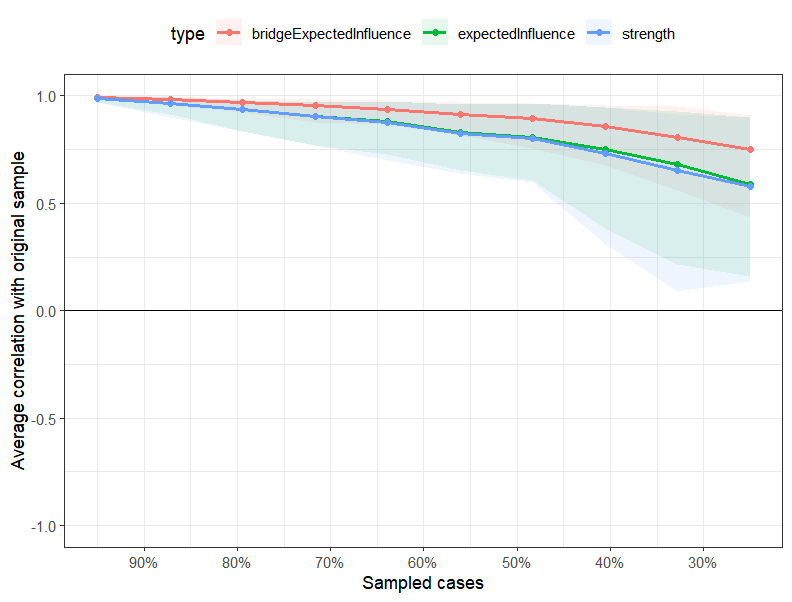
**

**Figur**S-2 Stability Analysis Results (Part 2)
